# Editorial: Integrating plant metabolites into comprehensive approaches for disease management

**DOI:** 10.3389/fnut.2026.1784962

**Published:** 2026-01-27

**Authors:** Felix O. Omoruyi, Cliff K. Riley, Lowell L. Dilworth

**Affiliations:** 1 Department of Health Sciences, Texas A&M University, Corpus Christi, TX, United States; 2 Hazardous Substances Regulatory Authority, Kingston, Jamaica; 3 Department of Pathology, The University of the West Indies, Mona, Jamaica

**Keywords:** bioactive compounds, flavonoids, inflammation, metabolic syndrome, oxidative stress, plant metabolites

Plant secondary metabolites have moved from the periphery of traditional nutrition and ethnopharmacology into the mainstream of biomedical and translational research. Accumulating evidence now positions these bioactive compounds as important modulators of metabolic, inflammatory, oxidative, and immune pathways that are associated with many chronic diseases. Derived from organisms ranging from simple algae to complex gymnosperms, plant metabolites exert hypolipidemic, hypoglycemic, antioxidant, anti-inflammatory, antimicrobial, and antitumor effects. Their broad biological activity, chemical diversity, and relative accessibility make them potential candidates for inclusion in disease management strategies that combine diet, lifestyle modification, and conventional therapeutics.

This Research Topic was conceived to advance understanding of how plant metabolites can be practically and scientifically integrated into disease prevention and management. The six peer-reviewed contributions, comprising systematic reviews and experimental research, demonstrate how plant-derived compounds can be used to prevent disease, support management strategies, and thus promote overall health and a healthier future. Together, these works underscore a shift from viewing plant products as ancillary, folklore or empirical remedies to recognizing them as evidence-based components of comprehensive health care.

A recurring theme across authors' contributions is the role of dietary patterns enriched with plant bioactive constituents in mitigating metabolic dysfunction. Cheng et al. examined the impact of a structured dietary intervention on sleep quality in individuals with metabolic syndrome (MetS), a condition characterized by metabolic, inflammatory, and oxidative disturbances. Using a single-arm, self-controlled design, the authors demonstrated that dietary supplementation with plant-based protein and herbal infusion containing tartary buckwheat, goji berries, and other bioactive ingredients significantly improved sleep quality. These benefits were linked to reductions in body mass index, improvements in insulin resistance, and favorable glucose and lipid profiles, as well as decreased inflammation and oxidative stress markers. Their findings reinforce the concept that plant-rich dietary interventions can exert multisystem benefits that extend beyond metabolic endpoints to clinical outcomes, such as sleep quality, an often-overlooked determinant of cardiometabolic health.

*Cuminum cyminum* (cumin) has long been valued for its antidiabetic, anti-inflammatory, and antioxidant properties, making it a plausible adjunct in the management of metabolic syndrome. Nevertheless, evidence from previous studies has been inconsistent, limiting clear interpretation. Liu et al. in their meta-analysis synthesizes randomized controlled trials (RCTs) data to examine the effects of cumin supplementation on MetS indices in adults with metabolic disorders. Their analyses demonstrated that cumin supplementation significantly improved several MetS indices. Heterogeneity was however evident across outcomes, likely due to variations in study populations, dosages, and intervention durations. Overall, this meta-analysis supports a potential role for cumin supplementation in improving glycemic control, lipid profile, and central adiposity among adults with metabolic disorders. While these findings are promising, the observed heterogeneity and limited number of high-quality trials underscore the need for larger, well-designed RCTs to establish efficacy, optimal dosing, and define target populations.

Beyond metabolic disease, plant metabolites also show potential to modulate fundamental biological processes involved in aging. Yang et al. explored the antioxidant and longevity-promoting effects of white tea extract in *Drosophila melanogaster*. Rich in flavonoids, white tea showed potent antioxidant activity *in vitro* and significantly extended lifespan in both male and female fruit flies. Longevity benefits were associated with enhanced antioxidant defense as evidenced by reduced lipid peroxidation, increased activities of superoxide dismutase and catalase, and upregulation of antioxidant genes. Although derived from a model organism, these findings provide mechanistic support for the role of dietary polyphenols in delaying age-related functional decline and highlight the need to further characterize specific compounds responsible for these effects and potential use in humans.

The importance of chemical complexity and synergism among plant metabolites was further emphasized in the comprehensive review by Peng et al., which reexamines coffee as a functional nutritional intervention rather than merely a stimulant beverage. Coffee contains a diverse array of bioactive compounds, including alkaloids, polyphenols, diterpenes, and Maillard reaction products, that interact within a complex regulatory network. The authors elucidated evidence linking these compounds to neuroprotection, metabolic regulation, antioxidant defense, and anti-inflammatory activity. By elucidating multi-target molecular mechanisms, the review redefines coffee as a dynamic, bio-synergistic system whose health effects may depend on the interplay between its natural chemical complexity and human biological variability.

Innovation at the interface of plant biology and nutritional science has given rise to the emerging field of plant-derived exosome-like nanoparticles (PDENs). Che et al. provide a systematic analysis of PDENs, which are enriched with plant proteins, lipids, nucleic acids, and secondary metabolites and exhibit cross-species regulatory capabilities. These nanoparticles can interact with mammalian cells and gut microbiota, modulate inflammatory signaling pathways, and serve as carriers for targeted delivery of bioactive compounds. While challenges related to scalability, stability, and safety remain, PDENs represent a promising frontier in functional food development, with potential applications in precision nutrition and anti-inflammatory therapies.

The therapeutic relevance of plant metabolites extends into oncology, as demonstrated by Ezcurra-Hualde et al., who investigated anthocyanins derived from *Hibiscus sabdariffa* in murine cancer models. The major anthocyanins, delphinidin-3-sambubioside and cyanidin-3-sambubioside exhibited potent antioxidant and antiproliferative effects in fibrosarcoma and colon carcinoma cell lines. Intratumoral administration *in vivo* significantly inhibited tumor growth and enhanced immune cell recruitment, particularly when combined with doxorubicin. These findings support the potential of specific plant flavonoids as adjunctive agents that can modulate the tumor microenvironment and enhance the efficacy of conventional chemotherapy.

Collectively, the studies in this Research Topic highlight the versatility of plant metabolites as modulators of health and disease. Whether through dietary optimization, functional beverages, nanoparticle-mediated delivery systems, or targeted therapeutic applications, plant-derived compounds offer multi-layered benefits that may align well with the complexity of chronic diseases. However, these contributions also emphasize the need for rigorous mechanistic studies, standardized formulations, and translational research to bridge experimental findings with clinical applications.

As lifestyle and age-related conditions increasingly drive global disease burdens, incorporating plant metabolites into comprehensive management strategies may offer a sustainable, biologically plausible approach to improving health outcomes. Embracing the synergistic nature of plant bioactive constituents, future research and clinical applications can better harness their full potential in disease prevention, management, and healthy aging.

